# Mental Health Apps Implemented in the Workplace: Scoping Review of Trends and Gaps in Evaluation Research

**DOI:** 10.2196/57046

**Published:** 2026-03-31

**Authors:** Sheila Addanki, Luciana Macedo, Joy MacDermid, Sandra Moll

**Affiliations:** 1 University of British Columbia, Vancouver, BC, Canada; 2 School of Rehabilitation Science, Faculty of Health Sciences, McMaster University, 1400 Main Street West, Hamilton, ON, L8S 1C7, Canada, 1 905-467-2155; 3 Western University, London, ON, Canada

**Keywords:** mobile apps, digital health, mobile health, mHealth, mental health apps, workplace, workers, scoping review, user engagement, smartphones, eHealth, technology-based interventions

## Abstract

**Background:**

Technology-based solutions to support the mental health needs of workers are on the rise, as evidenced by the growing body of research related to e–mental health apps implemented with workers or within the context of workplaces. This expanding landscape of evidence related to mental health apps underscores the necessity of summarizing and consolidating the different ways in which studies are evaluating real-world technology-based interventions in a complex setting such as a workplace.

**Objective:**

The aim of this scoping review is to summarize the growing body of evidence evaluating mental health apps with workers. Specific research questions include “What types of mental health apps are studied?” “With whom are they being evaluated?” and “What outcomes are being tracked with what tools?”

**Methods:**

The scoping review followed 5 stages: specifying the research question, identifying relevant literature, selecting studies, extracting data, and synthesizing the findings. The search strategy was applied across 6 databases (PsycINFO, Embase, MEDLINE, Cochrane Database, OVID Healthstar, and OVID Emcare) to identify relevant studies from January 2000 to August 2023.

**Results:**

From the 288 abstracts screened, 54 studies met the inclusion criteria for this review. Over two-thirds of the studies were randomized controlled trials. The studies included 44 different apps, comprising 23 structured self-guided apps, 15 unstructured self-guided apps, and 6 apps designed as adjuncts to other interventions. Evaluation approaches included examining user engagement and utilization, investigating the impact on users, and evaluating the implementation process. Most studies evaluated the impact on individual mental health–related outcomes as well as workplace-related outcomes.

**Conclusions:**

This scoping review provides a comprehensive overview of the ways in which studies are currently evaluating workplace mental health apps. The review highlights key trends and gaps in the existing research, noting that most studies focus on the effects of mental health apps on individual users, while only a limited number of studies explore how implementing such dynamic interventions within complex real-world settings (eg, workplaces) may influence their overall effectiveness. Future research should explore the implementation process to identify factors that promote and/or hinder the optimal use and impact of mental health apps for workers.

## Introduction

Mental health issues have a significant human and financial cost for society and workplaces. Poor mental health and loss of productivity are estimated to cost the global economy approximately US $1 trillion each year [1], and this figure is projected to rise, given the COVID-19 pandemic’s impact on workplace mental health. Rapid transitions and changes in workplace demands linked to the pandemic have led to an increase in reports of perceived stress, depression, and anxiety among working adults, particularly for working mothers, people of minority ethnicities, and those working in frontline positions [2-4]. Limited access to traditional mental health services, along with the longstanding stigma associated with seeking support, has led to a growing need to expand access to a range of mental health supports to address the unmet needs of the workforce and reduce the burden of mental health issues.

In recent years, there has been a growing interest in digital health technologies, including web-based and smartphone-based interventions as tools to address mental health problems. One of the main advantages of digital mental health interventions is their ability to overcome traditional barriers to accessing mental health care by providing on-demand access to quality mental health information and support [5,6]. For example, many people are reluctant to seek help due to the stigma associated with mental illness. Digital interventions, such as mental health apps, offer a private and anonymous way to access support without the fear of being judged. Digital interventions can also help overcome geographical barriers by providing access for people who live in remote or rural areas, where mental health services may be limited or nonexistent [5]. In addition, digital interventions can be accessed at any time and from anywhere, which can help overcome the time constraints that many working adults face when trying to access traditional mental health services. Mental health apps can be used across the full spectrum of mental health care, from prevention to management. For example, there are apps that help people assess and manage stress and anxiety, apps that provide support for people with depression, and apps that help people improve their well-being.

There is a growing body of research that supports the effectiveness of mental health apps in both the general and clinical populations [4,7]. A review of app-based mental health interventions found them to be effective in reducing symptoms of depression and anxiety [4,7], as well as improving mental health literacy and well-being [8] in both clinical and non-clinical populations [4,7-9]. However, a review of publicly available popular mental health apps found that only 2 out of the 6 mental health apps identified were empirically evaluated [10], underscoring the gap between mental health apps available publicly and those that are evaluated in empirical studies. There is an urgent need for high-quality studies in this field to explore mental health apps that are evidence-based, empirically supported, and scalable to broader users.

There is a growing interest in the use of mental health apps across different settings such as clinical, postsecondary, and community settings, and more recently in workplaces. Several reviews provide evidence to support that digital mental health interventions implemented in workplaces have positive effects on improving general mental health and workplace-related outcomes [11-13]. Studies have explored the effects of these interventions in improving well-being, symptoms of stress, and several workplace-related outcomes, such as work effectiveness or productivity and job satisfaction [11-13]. However, the majority of the studies in these reviews consisted of web-based interventions, with only a small percentage of them exploring mental health apps.

As the availability and use of mental health apps in workplace settings become more prevalent, there is a growing need to identify and synthesize the existing evidence related to their systematic evaluation with workers. In this paper, we aim to conduct a scoping review to synthesize and critically reflect on the body of evidence in this emerging field. Specifically, we will summarize and identify trends and gaps in the evaluation of mental health apps in workplace settings.

## Methods

### Study Design

We followed the 5 steps of scoping review methodology [14]: specifying the research question, identifying relevant literature, selecting studies, extracting data, and synthesizing the findings. The protocol for this review is registered on the Open Science Framework. We also adhered to the PRISMA-ScR (Preferred Reporting Items for Systematic Reviews and Meta-Analyses Extension for Scoping Reviews) guidelines (Checklist 1) to ensure consistent and transparent reporting [15]. To examine and summarize the breadth of evidence regarding the evaluation of mental health apps for working adults, we developed the following research questions based on the population, intervention, comparison, and outcome (PICO) framework:

1. How are mental health apps being evaluated with workers and/or in the context of a workplace?
2. What types of mental health apps are studied?
3. With whom are the applications being evaluated (workers or workplace sectors)?
4. What key variables are being assessed, and what tools are being used to track progress and impact?

### Inclusion Criteria

Studies were included in this review if they met the following inclusion criteria: (1) the primary study intervention was a mobile mental health app that was tested by employees; (2) the study evaluated the effectiveness, implementation, impact, or use of the mental health app; (3) study participants included workers currently employed in any field and/or implemented in the context of a workplace; (4) the study included any quantitative or qualitative evaluation design; (5) the study was published in a peer-reviewed journal; and (6) the study was available in English. Studies were excluded if: (1) the intervention in the study was primarily a web-based or internet-based intervention (ie, not a mobile app) and (2) the study design only focused on app development (not evaluation) of a mental health app for workers. Conference proceedings and gray literature were not included, as the key focus of this review was to present a summary of published peer-reviewed studies that systematically evaluated mental health apps.

### Search Strategy

We searched the following electronic databases: PsycINFO, Embase, MEDLINE, Cochrane Database, OVID Healthstar, and OVID Emcare for peer-reviewed studies from January 2000 to August 2023. A comprehensive search strategy, including search terms, subject headings, and syntax requirements for the databases, was adapted from a previous systematic review of digital mental health interventions for the workplace [11] and was modified in consultation with a health science librarian. Search terms included a combination of constructs such as “Mental Health,” “Workplace,” and “Mobile Application” (Multimedia Appendix 1). The search was conducted from May 2023 to August 2023.

### Study Screening

Data screening and extraction were carried out by 2 independent reviewers (SA and ML) using Covidence systematic review software (Veritas Health Innovation), an online tool that supports screening and data extraction for reviews, to facilitate a systematic screening process. All studies were screened independently by 2 reviewers, and any discrepancies in study selection were discussed in a review meeting. A third reviewer, SM, was consulted to resolve any disagreements between the 2 primary reviewers and to improve clarity in decision-making when necessary.

### Data Extraction and Analysis

Data extraction was an iterative process designed to develop and refine a data charting sheet in Microsoft Excel to ensure all relevant data were captured comprehensively. The fields of data extracted included study characteristics such as the year, country of publication, study design, workplace context or industry where the study was conducted, intervention characteristics, timeframe, and evaluation approaches including both assessment tools and outcomes explored in the study. The initial extraction chart was piloted with 5 studies and was later revised to capture additional information relevant to our research questions. Analysis of these data included a summary of the study characteristics, types of app interventions, study populations, and approaches to evaluation (variables and evaluation tools). Descriptive summaries of the trends in each category will be outlined.

## Results

### Overview

**Figure 1.**
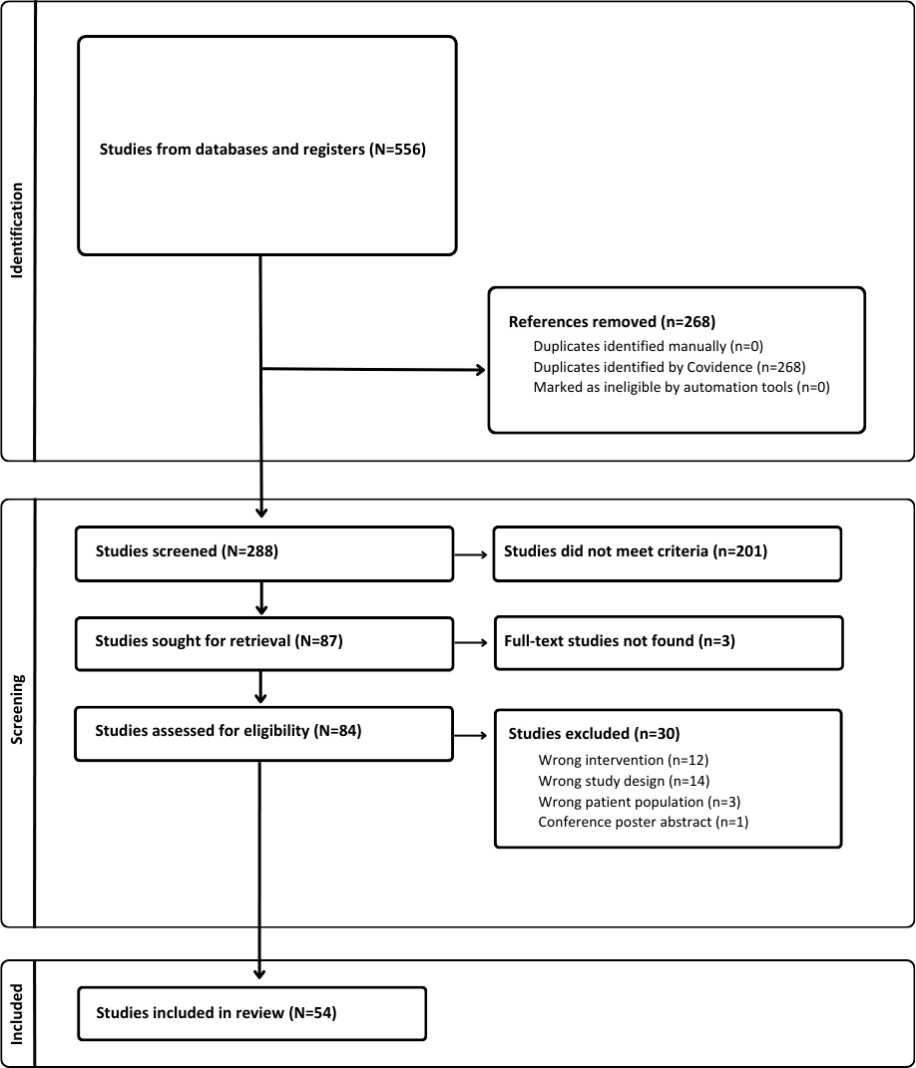
PRISMA (Preferred Reporting Items for Systematic Reviews and Meta-Analyses) flowchart of the study screening process.

We searched 6 electronic databases, identifying 288 studies after the removal of duplicates. After screening titles and abstracts, 85 studies were deemed eligible for full-text screening. Subsequently, 54 studies met our inclusion criteria for being included in this scoping review (Figure 1). Data from these studies were extracted and charted to generate a comprehensive summary of the breadth and trends in the body of evidence of mental health app evaluation in the workplace. Of these 54 studies, 11 were protocols for studies in progress; hence, no results were available for these studies at the time of the review. Upon careful consideration of the protocols and clinical trial registrations, we were able to gather relevant information related to the key outcomes being evaluated and the processes by which the studies were being conducted, which was deemed sufficient to answer our main research question: “How are mental health apps being evaluated with workers and/or in the context of the workplace?” As a result, we decided to report these ongoing studies in our review.

### Study Characteristics

Among the 54 studies included, approximately two-thirds were randomized controlled trials (RCTs; protocols or trial registrations n=11 [16-26]; completed RCTs n=21 [27-48]; pilot RCTs n=3 [49-51]). A smaller subset of studies adopted nonrandomized methods such as pre-post nonexperimental designs (n=13) [52-63], with even fewer studies using mixed methods (n=5) [64-67] and qualitative designs (n=1) [68]. Over two-thirds of the studies (n=37) were published after the onset of the COVID-19 pandemic, reflecting accelerated remote work and rapid digital transformation in workplaces, with over 26% (n=14) of the studies on this topic published in the year 2022 alone. Table 1 outlines a summary of the key characteristics of all included studies.

**Table 1.** Summary of study characteristics that are leading to significant shortages.

Characteristics	Studies per characteristic (n)
Manuscript availability	Full text (43) Ongoing (registered) clinical trials or protocols (11)
Study research design	Protocols (11) Pilot RCT^a^ (3) RCT (22) Cross-sectional pre- or postdesign (12) Mixed method studies (5) Qualitative (1)
Year of publication	2010 (1) 2013 (1) 2014 (1) 2016 (2) 2018 (8) 2019 (5) 2020 (9) 2021 (7) 2022 (13) 2023 (7)
Location of study	United States (13) Australia (12) United Kingdom (4) South Korea (5) Finland (3) Japan (2) Brazil (1) Europe (2) Italy (1) Sweden (1) Germany (1) Spain (1) Canada (1) Malaysia (1) Netherlands (1) Slovenia (1) Taiwan (1) Vietnam (1) England (2)
Organizational sector	Across multiple industries (7) Health care and social services (23) Information and technology (4) Education (2) Agriculture (1) Construction (1) Apprentice (1) Large corporations or consumer-based Industry (5) Unspecified (10)
Focus of intervention	Structured self-guided (23) Unstructured self-guided (15) Adjunct to other interventions (6)

a RCT: randomized controlled trial.

### Types of Mental Health App Interventions

A total of 44 unique apps were evaluated across the 54 studies included in this review. There was considerable variability across these apps in terms of their therapeutic approach, goals, functionalities, target population, and intervention duration. Over half of the apps (23/44, 52%) included a programmatic structure with predetermined guidelines or prescribed instructions on how to use them, while 34% (15/44) were open-ended and user driven without a fixed guideline for use. Based on these distinctions, we broadly classified these apps as: (1) structured self-guided, (2) unstructured self-guided, and (3) an adjunct to other interventions. See Table 2 for an overview of the identified apps categorized by type, including proposed purpose and target population noted in the study.

**Table 2.** Overview of apps by type and study target population.

Name of app	Overview	Target population
Unstructured self-guided		
e-pD-Work intervention [16]	Self-guided modules to promote physical exercise, improve sleep, expand relationships, solve problems, improve communication, assertiveness, decision-making, and manage work stress.	General practitioners across multiple primary care centers
The Healthy Minds Program [28]	Meditation app focused on awareness, connection, insight, and a model of well-being.	Employees from district school system
Stress management app (no official name found) [31]	Mental health information and mindfulness strategies (eg, music meditation and breathing).	Nurses employed at college hospitals
Lift Intervention [44]	Self-directed mindfulness program with tips and contents for integrating mindfulness into daily life.	Nurses from COVID-19 units at a single hospital
InMind [48]	Mindfulness training, including measurement of heart rate variability with recommended content.	Employees in 3 organizations: legal, construction, and a PR^a^ firm
24alife app [49]	Customized activities for relaxation, exercise training, nutritional advice, and physiological responses to stress based on initial assessment of lifestyle habits	Employees from an Information and Communication Technologies company
Jibun kiroku (a self-record app) [52]	CBT^b^-based intervention focuses on self-monitoring and awareness and recording of negative thoughts, daily activities, and daily mood	Employees experiencing mild to moderate psychological distress
The Wellness Hub app [55]	Mental health–related content and resources, and links to local resources.	Employees from a large health system
Listen Leon [56]	Encourages employees to send anonymous strengths-oriented descriptive feedback to colleagues	Employees from 5 industries: retail, consulting, banking, human resources, and education
Stress management app (no official name found) [57]	Mental health and lifestyle assessment, daily lifestyle management, peer support, tools for searching nearby mental health professionals, and relaxation training	Employees from 3 national hospitals and health promotion centers
Healthy Outcomes at Work app [63,64]	Psychoeducation tailored to the needs of the organization (info re: well-being events, access to vocational rehabilitation assistant, option to provide feedback (via app) to management	Child and Family social workers and hospital staff
The Mood Map [65]	Enables daily mood monitoring (happiness, sadness, anxiety, and anger) at regular intervals. Therapeutic strategies include cognitive reappraisal exercises, breathing, and relaxation techniques.	Employees at a large corporation with high stress levels
Oiva (app available in Finnish only) [66]	Promotes stress management and well-being, based on ACT^c^, with short skills building sessions (visual or audio).	Employees from 2 information communication and technology organizations
Brightr app [69]	Monitoring of behavior, sleep, physical activity, nutrition, and shift work by providing tailored feedback based on responses to short in-app questionnaires and sleep and physical activity tracking.	Employees from an information and technology company
Structured self-guided		
Unmind App [18,41]	Six to eight 10‐20 minute self-guided sessions based on principles of CBT and ACT to manage and improve mental health and well-being. Users had 3 weeks to complete their allocated intervention and were sent a weekly intervention reminder message.	Full-time or part-time employees from a research recruitment platform
SOLAR^d^ [20]	Eight modules over 5 weeks focused on behavioral skill development or psychoeducation, delivered in animations, videos, activities, and notifications, that participants can self-complete at their own pace. Developed as an extension of in-person training.	Firefighters experiencing distress (scored between 7 and 18 on the 6-item Kessler Psychological Distress Scale
WorkingWell [22]	Designed for individuals with severe mental health issues to cope on the job. Users are asked to select up to three goals to work towards and on which to reflect in the coming week. Daily reminders of goals upon app opening and new goals can be chosen for each week.	Employees with severe mental illness using supported employment services
EMPOWER [23]	7-week access to intervention: (1) screening tools for stressful psychosocial working conditions, mental distress, and work function; (2) psychoeducational CBT modules to promote mental health and work functioning.	Employees from SME^e^ and public agencies
WEDiary^f^ [24]	Two-week intervention that facilitates daily self-monitoring of goals and achievements at work.	Employees across different organizations
Holidaily [25]	Daily prompts based on behavioral activation strategies designed to promote recovery behavior and mental detachment from work to extend the beneficial effects of vacation into their daily work life.	Employees who are awaiting to go on vacation
Headspace [26,27,40,68]	Daily guided 10-minute mindfulness practices or techniques including breathing, imagination, and body scan exercises focusing on different bodily sensations.	Staff from 2 emergency departments (NHS^g^), health care workers, and university employees
BetterLife [29]	Guided, 10 weeks (50 min/wk) self-help therapy informed by principles of CBT and problem-solving therapy for addressing stress, depression, anxiety, and sleep disorders.	Employees with elevated perceived stress (score of 14+ on the Perceived Stress Scale)
Well-being mobile app (official name not found) [30]	A 8-week program (4 lessons/wk) with content focused on techniques to reduce stress and promote well-being (breathing techniques, relaxation techniques, and guided meditation).	Female employees at a large private tertiary care hospital
The MoodHacker [32]	A 6-week intervention with sequenced content and prompts to track mood and activities daily. App tracks mood and positive activity planning, promotes cognitive restructuring, mindful self-awareness, gratitude expression, and identifying and using strengths.	Employees from several institutions recruited in partnership with EAP^h^
Smartphone resiliency training (no official name found) [33]	A 6-week intervention for monitoring and awareness for sleep and mood, happiness and positivity, energy, and focus, and productivity. Users were prompted to select a goal (one of the topics) to work on for the next few weeks.	Employees from a large research hospital and medical center
ABC Stress Management app [36]	Six-week (1 module/wk) intervention with two types of stress management apps—Program A, a free-choice multimodule program and Program B, a fixed-sequential order iCBT^i^ multimodule program.	Employees from a large public hospital (Vietnam)
ACT-based app [37]	Six weekly modules based on ACT principles to manage stress. Reflections accessible by a therapist who could send encouraging personal messages to the user.	Middle managers from private sector company
HeadGear [17,38]	30-day therapeutic intervention (1 challenge/day). Content is based on behavioral activation and mindfulness-based therapies. Features include mood monitoring and access to a tech helpline.	Employees from male-dominated industry partner organizations: agriculture, freight, and mining
Anchored app (adapted from HeadGear app) [45,51]	30-day intervention with daily tasks or “challenge” based on evidence-based techniques (behavioral activation, mindfulness, and coping skills training) delivered using videos, exercise, and goal setting to target stress.	Adults experiencing workplace stress from any industry
HeadGear Apprentice app [67]	30-day therapeutic intervention (1 challenge/day). Modifications to HeadGear include gamification, changes to navigation, and customized content for apprentice support.	Trainees enrolled in an apprenticeship program
Kelaa Mental Resilience app [39]	Four weeks of psychoeducational modules designed to reduce stress and promote well-being specifically in the workplace context by providing options for self-monitoring of behaviors, cognitions, and emotions.	Employees from public and private sectors from 3 countries
Foundations app [43]	Daily active use encouraged during first 2 weeks. User chooses one of 6 focus areas during onboarding (relaxation, sleep, anxious thoughts, feeling down, self-esteem, and stress), then relevant programs and activities are presented based on CBT, mindfulness-based CBT, relaxation techniques, and positive psychology.	Health care workers across health system
Mobile-based Stress Management Intervention [46]	Recommended use of 10 minutes twice a week for 6 weeks. Combination of 3 modules that include interventions for relaxation, self-management, and counseling.	White collar workers with elevated symptoms of perceived stress (Perceived Stress Scale-10≥22)
Calm app [47]	Recommend 10 minutes per day over 8 weeks. Incorporates mindfulness, breathing techniques, and body scans.	Employees of a large consumer electronics retailer
P4Well [50]	Three mobile apps: (1) Nokia Wellness Diary, (2) Firstbeat Mobile Coach (fitness training), and (3) SelfRelax (personalized 4-wk relaxation program).	Male working adults (25‐45 y) with exhaustion, stress symptoms, or sleeping problems
Naluri app [53]	16-week EAP offering personalized programming to promote behavior change (educational modules, health journal, and a habit tracker) and access to a team of professional health coaches via text-based messages and video calls.	Employees from an unspecified company in Malaysia
Shift app [54,60]	30-day intervention based on content and features of HeadGear app, modified to meet the needs of physician population.	Junior medical officers from 2 hospital sites
VA^j^ Mindfulness Coach App [61]	12 audio-guided mindfulness exercises on a range of techniques (eg, body or breath awareness, compassion, and mindful eating).	Nurses from psychiatric facility reporting burnout
Smartphone-based meditation app (No official name found) [62]	Ten days of 10-minute audio-recorded mindfulness sessions.	Firefighters from a large metropolitan city
Adjunct to other interventions		
MATESmobile program [21]	Supplements in-person training with a focus on reinforcing face-to-face training messages and enabling links to mental health support as needed.	Construction workers
Spire Stone wearable device and mobile app [34]	Brief modules for mindfulness training with monitoring of respiratory patterns and real-time biofeedback of physiological stress.	Employees from a large technology corporation across seven cities.
Dayzz [42]	Personalized sleep training app that is an extension to an online SHAW^k^ program.	Employees at a large health care organization.
Brain-sensing eyeglasses and a corresponding mobile app [58]	Eyeglasses collected EEG^l^ activity during mindfulness, transferring the data to the app. App provided real-time acoustic feedback from data to promote relaxation.	Professionals with top management duties at a public service company
Smartphone-delivered biofeedback training (no official name found) [35]	Self-managed protocol with videos containing guided meditation practices and real-time biofeedback.	Psychiatric ward nurses in 3 hospitals who experienced workplace violence or abuse

a PR: public relations.

b CBT: cognitive behavioral therapy.

c ACT: acceptance and commitment therapy.

d SOLAR: The Skills for Life Adjustment and Resilience.

e SME: small and medium enterprises.

f WEDiary: work engagement diary.

g NHS: National Health Service.

h EAP: employee assistance program.

i iCBT: internet-based cognitive behavioral therapy.

j VA: Veterans Affairs.

k SHAW: Sleep Health and Wellness.

l EEG: electroencephalogram.

Structured self-guided apps (n=23 apps) had a structured pathway in the form of programs or modules that users needed to complete, often in the form of modules, lessons, exercises, or activities. For instance, the HeadGear app (cited in 8 studies) is a 30-day intervention informed by mindfulness-based techniques that include daily challenges users are instructed to complete [17,38,45,51,54,60,67]. Headspace included daily guided 10-minute mindfulness practices or techniques [26,27,40,68]. Several studies noted that the app content was informed by evidence-based therapies, including behavioral activation and mindfulness [24,38,48,53,62,70], acceptance and commitment therapy [37,42,43,68], and principles of cognitive behavioral therapy [32,59], but varied widely in terms of the extent to which they adhered to evidence-based guidelines.

Unstructured self-guided apps (n=15) were more open-ended and provided users with the choice to select and engage with intervention components on demand. For instance, the Oiva app [66] consisted of information sessions on stress management and well-being based on the principles of acceptance and commitment therapy but had no prescribed guidance for engaging with the app. Many apps included meditation or relaxation exercises [28,31,35,44,48,49], self-monitoring tools (eg, mood, physical activity, and stress) [52,57,65,69], and/or links to resources [55,57].

Finally, there were 5 apps that were designed to be implemented in conjunction with other interventions. Three were used to supplement in-person training [21,34,42], and three included wearable devices linked to the mental health app [34,35,58]. One study, for example, combined a brief in-person mindfulness training followed by monitoring of respiratory patterns through a wearable device, with real-time biofeedback received through the app [34].

In terms of intervention duration, most studies (n=38) implemented a fixed intervention period, typically ranging from 2 to 8 weeks, with 4 weeks being the most common. Outcomes were generally assessed at baseline and at the end of the intervention, although some studies included longer follow-up periods, with the longest extending to 12 months [38].

### Study Populations

Participant demographics varied significantly across the studies. Some studies targeted the implementation of the intervention with specific gender groups, among individuals with symptoms of mental health issues and/or in specific workplace sectors. For instance, one study targeted male participants experiencing mild to moderate symptoms of stress and/or depression to assess the feasibility of a multicomponent, technology-based (P4Well) intervention [50]. Two other studies specifically recruited individuals from male-dominated industries to evaluate the effectiveness of a therapeutic app (HeadGear) [38,45]. One study focused on evaluating the effectiveness of a well-being and stress management app for working women [30].

Study participants worked in a range of industries, including health and social services (n=23), which largely aimed at implementing therapeutic apps, large corporations or consumer-based industries (n=5), information and technology (n=5), education (n=2), agriculture (n=1), apprenticeships (n=1), and construction (n=1). A subset of studies implemented their interventions across multiple industries (n=7), and a few studies did not explicitly specify the contextual setting (n=10) in which the research was being conducted.

### Approach to Evaluation

**Figure 2.**
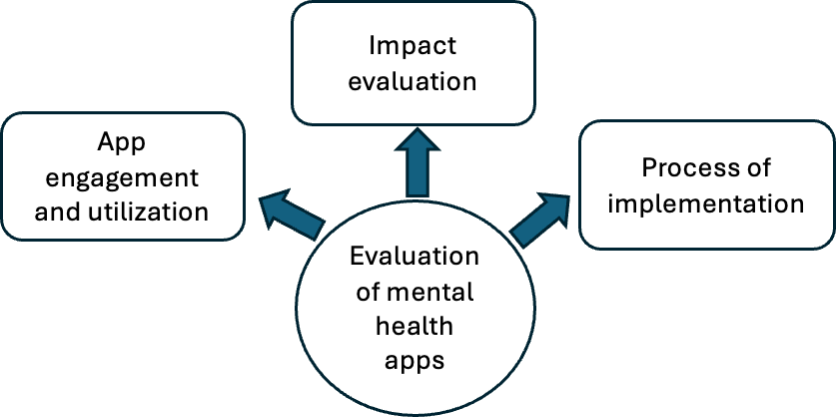
Three primary approaches to evaluating mental health apps in the workplace.

We identified three primary ways (Figure 2) in which studies evaluated mental health apps with workers: (1) app engagement and utilization; (2) impact evaluation (mental distress, mental health, and workplace outcomes); and (3) implementation process evaluation. The variables and tools within each approach are summarized in Multimedia Appendix 2.

#### App Engagement and Utilization

Most of the studies reported on users’ engagement with the intervention by tracking app usage patterns. App use ranged from simply tracking the number of downloads or logins to tracking the number of times specific features were used, completion of specific tasks, and tracking the amount of time spent in the app. In the structured interventions, app usage included tracking adherence to the prescribed protocols. The data was typically gathered through built-in analytics that highlighted aggregate usage patterns of the app, although some studies gathered subjective reports of app usage through survey or interview questions. There were at least a dozen studies that did not report app usage data.

User feedback was another common way of tracking engagement, noted in 22 (41%) of the studies. Typically, the follow-up evaluation included exploring perceptions of the app’s key features, such as usability, acceptability, quality, motivation to use the app, and overall satisfaction, and/or providing an opportunity to suggest improvements. Standardized measures, such as the Mobile App Rating Scale [70], were used in 5 studies [18,25,41,51,67]; the Systems Usability Scale [71] was used in 2 studies [22,32]; and the eHealth Impact Questionnaire was used in 1 study [16]. Other studies used more informal feedback approaches.

#### Impact Evaluation

Most of the research on the impact of mental health apps for workers focuses on multiple primary and secondary outcomes, ranging from mental distress to mental health and well-being, as well as specific work-related outcomes for individual app users. Table 3 highlights the most common outcomes that were evaluated, along with an overview of the tools that were used to track impact.

**Table 3.** Impact outcomes and measurement tools across studies.

Outcomes and variables	Most frequent measures used
Mental distress	
Symptoms of depression [16-20,23,25,29,31-35,38,40-42,44-47,50-55,57,60,62,67]	PHQ-9^a^ and PHQ-8 (n=16) DASS-21^b^ (n=4) Center for Epidemiologic Studies Depression Scale (n=3) Patient Reported Outcomes Measuring Scale (n=3) Beck Depression Inventory (n=2)
Symptoms of anxiety [16-18,20,23,28,29,31,32,34,38,41,43,45,46,49,51,52,54,55,60,62,65,67]	GAD-7^c^ and GAD-2 (n=16) STAI^d^ (n=2)
Psychological distress [21,37,43,50,52]	General Health Questionnaire (n=2) Kessler Psychological Distress Scale (n=2) General Symptom Index
Perceived stress [25-31,34,37,39,45,46,48,49,57,58,62,63,68]	PSS^e^ (n=16) Biological markers of stress (n=4)
Psychological trauma [20,55]	PCL-5^f^
Alcohol use [51,52,55,57]	AUDIT-C^g^ (n=2) The Daily Drinking Questionnaire (n=1)
Sleep [25,26,33,39,42,47,49]	Pittsburgh Sleep Quality Index (n=2) Insomnia Severity Index (n=2) Fatigue inventory Sleeping troubles from COPSOQ-II^h^
Mental health	
Well-being [17,18,23,27,28,30,31,33,38-41,43,45,48,51,52,55,56,63,66-68]	WHO^i^-5 Well-Being Index (n=12) Warwick-Edinburgh Mental Wellbeing Scale (n=7)
Quality of life [16,23,29,34,50]	Visual Analog Scale Euro Quality of Life Questionnaire World Health Organization Quality of Life CDC^j^ health-related quality of life metrics
Resilience [17,35,38,39,43,45,47,51,55,57]	Connor-Davidson Resilience Scale (n=3) Brief Resilience Scale (n=4) Resilience Scale (n=2) Korean Resilience Quotient
Self-compassion [28,33,40]	Self-Compassion Scale (n=2)
Life satisfaction [28,49,50,66]	5-item SWLS^k^ (n=2) Meaning in Life Questionnaire–Presence Subscale
Mindfulness [26-28,40,66]	Five Facets Mindfulness Questionnaire (n=3) Mindful Attention Awareness Scale
Social connections [28,57]	Social Readjustment Rating Scale Social connection (NIH^l^ Toolbox Loneliness Questionnaire)
Work-related outcomes	
Work stress [26,31,35,51,57]	SISQ^m^ Korean Occupational Stress Scale (n=2) Siegrist Job Strain Scale Occupational Stress Indicator-2
Burnout [25-27,33,40,44,45,49,50,61,62,68]	Maslach Burnout Inventory (n=8) Bergen Burnout Inventory (n=3)
Work engagement [22,24,26,29,36,46,56,63,66]	Utrecht Work Engagement Scale (9 items; n=7) Job motivation (job match survey and Multidimensional Work Motivation Scale; n=2)
Job satisfaction [16,22,63]	Self-report (n=3)
Work performance or productivity [17,23,32,38,39,41-43,45,47,51,56,60,63,67]	HPQ^n^ (n=6) The Work Productivity and Activity Impairment Questionnaire (n=3) WLQ^o^ Stanford Presenteeism Scale Productivity cost (count of absenteeism and presenteeism; n=2)
Working conditions [16,21,31,39,56,63,64]	MSIT^p^ (n=2) Job Content Questionnaire Korean-Emotional Labor scale Psychosocial safety climate Strengths Use and Deficit Correction scale
Other outcomes	
Mental health knowledge [21,32]	Author developed 14 multiple-choice scale GAT^q^ suicide awareness questionnaire
Help-seeking or health care utilization [21,23,42,43,47,54,60,72]	General Help Seeking Questionnaire (n=2) Intentions of help seeking Self-report of medical visits or help accessed (n=3)
Healthy behaviors or physical function [23,49,57]	IPAQ^r^ Fitness test (strength and flexibility) Exercise and diet patterns
Cognitive function [28,50,58]	Neurocognitive efficiency tests Psychological flexibility (Acceptance and Action Questionnaire-2) Perseverative thinking and Drexel Defusion Scale

a PHQ: Patient Health Questionnaire.

b DASS-21: Depression, Anxiety, and Stress Scale–21.

c GAD: Generalized Anxiety Disorder.

d STAI: State-Trait Anxiety Inventory.

e PSS: Perceived Stress Scale.

f PCL-5: Posttraumatic Stress Disorder Checklist for *Diagnostic and Statistical Manual of Mental Disorders, Fifth Edition*.

g AUDIT-C: Alcohol Use Disorders Identification Test–Concise.

h COPSOQ-II: Copenhagen Psychosocial Questionnaire-II.

i WHO: World Health Organization.

j CDC: Centers for Disease Control and Prevention.

k SWLS: Satisfaction with Life Scale.

l NIH: National Institutes of Health.

m SISQ: Single-Item Stress Question.

n HPQ: Health and Work Performance Questionnaire.

o WLQ: Work Limitations Questionnaire.

p MSIT: Management Standards Indicator Tool.

q GAT: General Awareness Training.

r IPAQ: International Physical Activity Questionnaire.

Impact on mental distress was the most common outcome, evaluated in 42 of the 54 (78%) studies (Table 3). Symptoms of depression were evaluated in 30 (56%) of the studies, often using standardized tools such as the Patient Health Questionnaire-9 [73]. Symptoms of anxiety were evaluated in 24 (44%) of the studies, many using the Generalized Anxiety Disorder (GAD-2 or GAD-7) [74]. Perceived stress was another common outcome measure used in 18 of the 54 (33%) studies, with tools such as the Perceived Stress Scale [75]. It is interesting to note that four of the studies incorporated biological indicators of stress [49,58,62]. Other measures of change in mental distress included tools to track symptoms of trauma, alcohol use, and sleep or insomnia.

Impact on mental health and well-being was another common outcome, evaluated across 34 (63%) studies. The primary outcome was the assessment of impact on well-being (n=24, 44%), using standardized tools such as the World Health Organization–5 (WHO-5) Well-Being Index [76] or the 14-item Warwick-Edinburgh Wellbeing Scale [77]. Other well-being outcomes included resilience (n=10), life satisfaction (n=7), quality of life (n=5), mindfulness (n=5), self-compassion (n=3), and social connection (n=2). These measures are congruent with many of the tools that focused on mindfulness and psychoeducation for coping with stress.

Impact on work-related outcomes was examined in 37 (68%) studies. Common work-related outcomes included work productivity and performance, burnout, job satisfaction, work engagement, and subjective perceptions of work stress and working conditions. Work performance outcomes, noted in at least 15 (30%) studies [17,23,32,38,39,41-43,45,47,51,56,60,63,67], included standardized tools such as the Work Limitations Questionnaire, the WHO Health and Work Performance Questionnaire, and the Work Productivity and Activity Impairment Questionnaire. Many of these tools included presenteeism or absenteeism measures, but several studies evaluated these separately [23,40,43,45,63,67], with some noting the productivity cost for organizations [23,47,67]. Burnout was another relatively common outcome noted in 12 (22%) of the studies [25-27,33,40,44,45,49,50,61,62,68], and evaluated using tools such as the Maslach Burnout Inventory or Bergen Burnout Inventory. Positive measures of job satisfaction were noted in four studies, often tracked through self-report [16,19,22,63]. Work engagement was another outcome noted in 9 (17%) studies [22,24,26,29,36,46,56,63,66], typically measured by tools such as the Utrecht Work Engagement Scale. In addition to work outcomes, 14 (26%) studies evaluated whether using a mental health app could change users’ perceptions of their working conditions including perceived work stress [25,26,31,35,51,57], job demands or strain [16,31,36], psychosocial climate [21,23], and perceived social support, including support from leaders and co-workers [39,43,63,64].

Other outcomes used to track impact included changes in mental health knowledge [21,32], cognitive function [28,50,58], physical function [49], or engagement in healthy behaviors related to diet and exercise [23,57]. Help-seeking or health care utilization was another outcome noted in 7 (13%) studies [21,23,42,43,47,54,60], particularly with apps that promoted connection to mental health resources. It is interesting to note that very few studies evaluated behavior change.

#### Process of Implementation

While most studies evaluated the overall effectiveness of the technology on individual or work-related outcomes, only a limited number of studies (n=5) focused on evaluating how the process of implementation affected these outcomes. Protocol studies included in this review proposed examining the relationship between the extent of app usage [19] in terms of its influence on the overall effectiveness of the intervention and understanding strategies needed to initiate and promote the use of the app [22]. Implementation outcomes in 3 other studies involved the evaluation of individual, contextual, and implementation-related barriers and facilitators that influenced the uptake, use, and overall impact of the mental health intervention [66,68,69]. Process measures were incorporated in order to generate recommendations for optimizing the utilization and effectiveness of the mental health technology.

## Discussion

### Principal Findings

This scoping review identified 54 studies (43 completed RCTs and 11 protocols) evaluating mobile health (mHealth) applications in workplace contexts. The main trends in the research were (1) research on workplace mental health apps has expanded rapidly in recent years, particularly following the pandemic; (2) the majority of studies were conducted in health and social services, with few in male-dominated industries; (3) about half of the 44 distinct apps adopted a structured, self-guided approach with modules or activities for the user to complete, another third were unstructured apps to be used as needed, and 11% of the apps were designed to be used in conjunction with a wearable sensor or web-based program; and (4) commonly evaluated outcomes included mental distress (including perceived stress, anxiety, and depression), mental wellness (well-being and resilience), and workplace-related outcomes (burnout, workplace engagement, work productivity, and performance), with limited studies on the process of implementation. Together, these findings highlight important gaps in both the design and evaluation of workplace mental health apps, underscoring the need for greater use of implementation science frameworks to guide future research.

### Study Characteristics  

The results of this review profiled 43 completed studies and 11 study protocols that evaluate the impact of mHealth interventions on a working population. A previous systematic review, published in 2019, on e–mental health interventions in the workplace identified 22 studies, but only 3 studies included apps as a primary intervention [78]. The pandemic and post-pandemic era have precipitated a rapid increase in app-related research with workers (41 studies in the past 5 years). This may reflect a shift to mHealth approaches to support the growing mental health needs of workers. The number of completed randomized clinical trials (24/54, 44%) is promising, reflecting high-quality research designs to evaluate impact. Furthermore, this review included 11 trial registrations or published protocols, reflecting that the research is ongoing. The rise of digital technology and associated research on mental health apps for workers speaks to the importance of summarizing current evaluation practices and gaps in the evidence.

### Types of Mental Health Applications

The review also highlighted diversity in the nature of mHealth interventions, with 44 different types of unique applications across the mental health spectrum, from promoting wellness to managing illness. In fact, several studies included wearable devices linked to the app, which may reflect a new technology trend. As noted in other review studies of mobile apps in the workplace, many were based on principles of cognitive behavioral therapy or mindfulness, combining information sharing with self-monitoring and self-management activities [11-13,79]. It is interesting to note, however, that even in the structured apps, the mechanisms of action were not consistently explained. There were a few studies that explicitly cited theories such as behavioral activation and cognitive behavioral approaches, but many of the app tools and techniques were not necessarily embedded within a psychotherapeutic protocol. This is a significant gap since explicit links between app design and expected outcomes are important aspects of evidence-based interventions [80].

Over half of the studies featured structured, self-guided apps with specific modules or activities that users were directed to complete, often over a 2 to 8-week period. Outcomes related to engagement, including completion of the activities, were more amenable to tracking “success” than apps that were less structured and available to use “as needed.” Given high attrition rates in app use, recommendations for optimizing engagement noted in the literature include providing “challenges” within the technology over short periods to build habits, providing regular “push notifications,” and connecting with an online coach or therapist [81]. Additional research is needed, however, to systematically evaluate the effect of these implementation strategies, and longitudinal research is needed to track the impact of these strategies over time [79,81]. Only a few apps (Headspace, Headgear, or Shift) had more than 2 studies examining their effectiveness, reflecting the need for more robust evaluation.

### Study Participants

Although studies in this review were conducted across a range of sectors, the highest proportion (23/54, 42%) was in health and social services. Given the mental health crisis noted in health and social services that is leading to significant shortages in staffing [44,68], these studies indicate promising efforts to evaluate innovative strategies to support the mental health of this group of workers. Studies in other sectors were more limited, although there were several in service-based sectors (education and information technology), with comparatively fewer studies in male-dominated industries such as construction. Unfortunately, 10 of the studies did not specify the workplace context where the app was implemented, making it difficult to identify relevant recommendations for specific workplace sectors. Given the importance of the workplace context, future comparative research is needed to explore how mobile apps function differently across industry sectors and organizations. Furthermore, many studies included in this review lacked sufficient reporting details regarding the intervention, research setting, and evaluation plan, making it challenging for future reproducibility, comparability, and dissemination. There are guidelines, such as the 16-item mERA (mHealth Evidence Reporting and Assessment) checklist, that can be used to improve the reporting quality of interventions and support replicability of research [82]. This checklist provides comprehensive recommendations for study authors on adequately describing the content, context, and technology infrastructure to implement the mobile health technology with a specific population group [82].

### Key Evaluation Variables and Tools

The review highlights 3 different categories of evaluations conducted by studies exploring mental health apps. User engagement and feedback were important outcomes tracked across many studies, either objectively through app metrics or subjectively through a recall of usage patterns. Feedback about usability, acceptability, and relevance was tracked qualitatively but also through standardized measures such as the Mobile Application Rating Scale [70] and the System Usability Scale [71]. Tracking user perceptions, engagement, and interaction with mental health apps is critical in establishing a relationship between usage (dose) and desired outcomes (response) [79]. Individual studies varied in their operational definitions of these constructs along with how they were measured, ranging from objective measures of usage (frequency and duration of use) that are passively collected from within the app to subjective measures of user ratings of usefulness and satisfaction, making it difficult to conduct cross-study comparisons.

Currently, there is no consensus on what constitutes optimal engagement for a mental health app to produce desired effects [83,84]. One study exploring different types of app use, features, and their effect on outcomes found that the right combination of different features (ie, learning, goal setting, and self-tracking) and their use at a moderate level could lead to desired outcomes, thereby refuting the common belief that higher usage always leads to better outcomes [85]. Collectively, each of the identified measures could be valuable in determining whether users who reported higher levels of usage, found the app useful, and were satisfied were more likely to engage with the intervention optimally, subsequently leading to significant improvements in outcomes. However, only 1 study protocol proposed assessing the link between the dose (the extent to which an app needs to be used) and the response (a significant improvement in outcomes) [19].

In addition to assessing usage and engagement, user experience and feedback were typically assessed only in feasibility studies. Since apps require iterative updates and maintenance, assessing user experience and feedback over time is important when attempting to deploy them in a large-scale, real-world setting [84].

In terms of impact, it is interesting to note that there was a range of approaches to evaluation, ranging from impact on reducing mental distress (78%) to improving mental well-being (63%) or work-related outcomes (68%). Since all of the studies were conducted in the context of the workplace, these workplace-related outcomes may be important for organizational decision-makers who are considering the value of investing in mHealth technology. Although there were 37 studies that considered workplace outcomes, there was a wide range of tools to evaluate these outcomes, ranging from downstream measures of productivity and burnout to more upstream measures of work engagement, perceived working conditions, and job satisfaction. Although there is a growing body of evidence indicating that digital mental health interventions implemented in workplaces can improve work effectiveness and productivity [13], tracking these outcomes can be complex, considering that studies need to speculate when changes in these workplace performance outcomes will take place and embed appropriate follow-up time points to measure them [86]. Study designs need to clearly outline the components of the intervention and the mechanisms of change to define what constitutes proximal (short-term or immediate) outcomes and distal (long-term) outcomes and determine appropriate time points for the measurement of these outcomes.

Even when apps are designed to be evidence-based, this does not guarantee that the expected outcomes will occur. Evaluating a complex mental health intervention in the context of the workplace requires a multilevel approach, considering the implementation process as well as its impact [81,86]. While several studies in this review acknowledged the complexities of implementing a digital intervention in a workplace setting, very few systematically evaluated the process of implementation, and there appears to be a gap in the application of existing theories to systematically guide this inquiry.

Several implementation science theories and frameworks can be useful to guide further research in understanding and unpacking the complexities of implementing technology across different contexts and achieving large-scale adoption and sustained effects. For example, the Consolidated Framework for Implementation Research (CFIR) provides a summary of evidence-based constructs across 5 domains: the intervention, the individual user, the inner context, the outer context, and the process of implementation [87]. These constructs can help identify and explain the facilitators and barriers to the uptake and impact of the intervention [87]. Another similar framework, called the nonadoption, abandonment, scale-up, spread, and sustainability (NASSS) framework [88], highlights six key domains that can be used to guide researchers in evaluating the success of an mHealth intervention implemented in an organizational context. It considers the characteristics of (1) the condition being addressed (eg, mental health), (2) the technology-based intervention, (3) the adopters of the technology, (4) the value proposition for the users, (5) the organization where the technology is being implemented, and (6) the wider system or society. The NASSS framework also reflects on the complex and dynamic interaction between the 6 domains over time, which can help answer questions related to users’ engagement with apps over time [88]. Systematic evaluation of the implementation process can facilitate exploration of key facilitators and barriers that influence the uptake and effectiveness of the intervention. While traditional health care interventions typically emphasize evaluation of their effects on key outcomes, mental health app–based interventions necessitate the examination of the different ways in which users engage with the intervention as well as exploring other implementation-related and contextual factors that influence the uptake and overall impact [79-81,83,86].

### Limitations

To our knowledge, this review is one of the first to summarize current research regarding approaches to evaluating mental health apps specifically in the context of the workplace. While a broad objective was established at the onset of the review in order to sufficiently capture all relevant studies, we restricted our search to studies that were published in English to ensure feasibility in terms of timelines and resources. Another limitation was the focus only on descriptions of the evaluation approaches outlined in the studies. There was no formal assessment of the study quality or synthesis of study findings. Future systematic reviews are needed to critically evaluate the current research evidence to identify the impact of mental health apps, and to profile what works for whom and in what context. This is particularly important to assist organizational leaders, policymakers, and stakeholders in deciding how to invest in and optimize the use of mental health apps with frontline workers.

### Recommendations

Results from this study informed the following recommendations for future research regarding app-based mental health interventions in the workplace. First, future research should improve transparency and consistency in the reporting of studies by incorporating guidelines such as the CONSORT-EHEALTH (Consolidated Standards of Reporting Trials of Electronic and Mobile Health Applications and Online Telehealth) [89]. Second, evaluation studies need to identify and measure appropriate outcomes by examining the congruence between intervention goals and outcomes when exploring the effectiveness of mental health apps. Third, there is a need for longitudinal studies to assess the long-term effects of mental health apps, particularly when evaluating their impact on workplace outcomes that may require a longer duration to detect meaningful changes. Fourth, long-term effects of mental health apps, particularly when evaluating their impact on workplace outcomes that may require longer duration to detect meaningful changes. Fifth, there is a need to investigate strategies for integrating mental health apps into existing workplace settings by considering organizational factors and fostering an implementation science lens. Applying implementation theory can generate new insights about what works for whom and in what context. Sixth, there is a need to investigate strategies for integrating mental health apps into existing workplace settings by considering organizational factors and fostering an implementation science lens. Applying implementation theory can generate new insights about what works for whom and in what context. Finally, a future systematic review is needed to synthesize and appraise current evidence regarding the effectiveness of mental health apps for workers.

### Conclusions

In conclusion, the past decade has seen tremendous advancements in the field of mHealth innovation, including its application in the context of the workplace. Traditional experimental approaches to evaluating mHealth interventions, however, may not be sufficient to capture the complexities of the facilitators and barriers that shape the adoption of the intervention and the sustainability of its effects within diverse workplaces. This methodological gap calls for research in this field to embrace new implementation science methodologies to truly capture the nuances of technology adoption and reliably measure its effectiveness over time.

## Supplementary material

10.2196/57046 Multimedia Appendix 1 Search strategy.

10.2196/57046 Multimedia Appendix 2 Study summary table.

10.2196/57046 Checklist 1 PRISMA-ScR checklist.

## Abbreviations

CFIR: Consolidated Framework for Implementation Research
CONSORT-EHEALTH: Consolidated Standards of Reporting Trials of Electronic and Mobile Health Applications and Online Telehealth
mERA: mHealth Evidence Reporting and Assessment
mHealth: mobile health
NASSS: nonadoption, abandonment, scale-up, spread, and sustainability
PICO: population, intervention, comparison, and outcome
PRISMA-ScR: Preferred Reporting Items for Systematic Reviews and Meta-Analyses Extension for Scoping Reviews
RCT: randomized controlled trial
WHO: World Health Organization

## References

[1] The Lancet Global Health (2020). Mental health matters. Lancet Glob Health.

[2] Brooks SK, Webster RK, Smith LE (2020). The psychological impact of quarantine and how to reduce it: rapid review of the evidence. Lancet.

[3] Tulk C, Bartram M, Leslie K, Atanackovic J, Chamberland-Rowe C, Bourgeault IL (2023). The impact of COVID-19 on the mental health and substance use health (MHSUH) workforce in Canada: a mixed methods study. Hum Resour Health.

[4] Rauschenberg C, Schick A, Hirjak D (2021). Evidence synthesis of digital interventions to mitigate the negative impact of the COVID-19 pandemic on public mental health: rapid meta-review. J Med Internet Res.

[5] Olff M (2015). Mobile mental health: a challenging research agenda. Eur J Psychotraumatol.

[6] Watts SE, Andrews G (2014). Internet access is not restricted globally to high income countries: So why are evidenced based prevention and treatment programs for mental disorders so rare?. Asian J Psychiatr.

[7] Firth J, Torous J, Nicholas J (2017). The efficacy of smartphone-based mental health interventions for depressive symptoms: a meta-analysis of randomized controlled trials. World Psychiatry.

[8] Bakker D, Kazantzis N, Rickwood D, Rickard N (2016). Mental health smartphone apps: review and evidence-based recommendations for future developments. JMIR Ment Health.

[9] Seegan PL, Miller MJ, Heliste JL, Fathi L, McGuire JF (2023). Efficacy of stand-alone digital mental health applications for anxiety and depression: a meta-analysis of randomized controlled trials. J Psychiatr Res.

[10] Wasil AR, Palermo EH, Lorenzo-Luaces L, DeRubeis RJ (2022). Is there an app for that? A review of popular apps for depression, anxiety, and well-being. Cogn Behav Pract.

[11] Stratton E, Jones N, Peters SE, Torous J, Glozier N (2021). Digital mHealth interventions for employees: systematic review and meta-analysis of their effects on workplace outcomes. J Occup Environ Med.

[12] Howarth A, Quesada J, Silva J, Judycki S, Mills PR (2018). The impact of digital health interventions on health-related outcomes in the workplace: a systematic review. Digit Health.

[13] Carolan S, Harris PR, Cavanagh K (2017). Improving employee well-being and effectiveness: systematic review and meta-analysis of web-based psychological interventions delivered in the workplace. J Med Internet Res.

[14] Arksey H, O’Malley L (2005). Scoping studies: towards a methodological framework. Int J Soc Res Methodol.

[15] Tricco AC, Lillie E, Zarin W (2018). PRISMA extension for scoping reviews (PRISMA-ScR): checklist and explanation. Ann Intern Med.

[16] Bellón JA, Rodríguez-Morejón A, Conejo-Cerón S (2023). A personalized intervention to prevent depression in primary care based on risk predictive algorithms and decision support systems: protocol of the e-predictD study. Front Psychiatry.

[17] Deady M, Johnston DA, Glozier N (2018). A smartphone application for treating depressive symptoms: study protocol for a randomised controlled trial. BMC Psychiatry.

[18] Economides M, Male R, Bolton H, Cavanagh K (2023). Feasibility and preliminary efficacy of app-based audio tools to improve sleep health in working adults experiencing poor sleep: a multi-arm randomized pilot trial. Sleep.

[19] Kosenkranius MK, Rink FA, de Bloom J, van den Heuvel M (2020). The design and development of a hybrid off-job crafting intervention to enhance needs satisfaction, well-being and performance: a study protocol for a randomized controlled trial. BMC Public Health.

[20] Metcalf O, Gibson K, Fredrickson J, Finlayson-Short L, Varker T, O’Donnell M (2023). Design, development and randomised controlled trial protocol of a smartphone-delivered version of “SOLAR” for emergency service workers to manage stress and trauma. BMJ Open.

[21] Milner A, King TL, Scovelle AJ (2019). A blended face-to-face and smartphone intervention for suicide prevention in the construction industry: protocol for a randomized controlled trial with MATES in Construction. BMC Psychiatry.

[22] Nicholson J, Wright SM, Carlisle AM (2018). Pre-post, mixed-methods feasibility study of the WorkingWell mobile support tool for individuals with serious mental illness in the USA: a pilot study protocol. BMJ Open.

[23] Olaya B, Van der Feltz-Cornelis CM, Hakkaart-van Roijen L (2022). Study protocol of EMPOWER: a cluster randomized trial of a multimodal eHealth intervention for promoting mental health in the workplace following a stepped wedge trial design. Digit Health.

[24] Tokita M, Kobayashi S, Miyanaka D (2025). Effects of a smartphone-based positive reflection diary on work engagement among Japanese workers: randomized controlled trial. JMIR Mhealth Uhealth.

[25] Smyth A, de Bloom J, Syrek C (2020). Efficacy of a smartphone-based intervention—“Holidaily”—promoting recovery behaviour in workers after a vacation: study protocol for a randomised controlled trial. BMC Public Health.

[26] Rich RM, Ogden J, Morison L (2021). A randomized controlled trial of an app-delivered mindfulness program among university employees: effects on stress and work-related outcomes. Int J Workplace Health Manag.

[27] Xu HG, Eley R, Kynoch K, Tuckett A (2022). Effects of mobile mindfulness on emergency department work stress: a randomised controlled trial. Emerg Med Australas.

[28] Hirshberg MJ, Frye C, Dahl CJ (2022). A randomized controlled trial of a smartphone-based well-being training in public school system employees during the COVID-19 pandemic. J Educ Psychol.

[29] Hwang H, Kim SM, Netterstrøm B, Han DH (2022). The efficacy of a smartphone-based app on stress reduction: randomized controlled trial. J Med Internet Res.

[30] Coelhoso CC, Tobo PR, Lacerda SS (2019). A new mental health mobile app for well-being and stress reduction in working women: randomized controlled trial. J Med Internet Res.

[31] Hwang WJ, Jo HH (2019). Evaluation of the effectiveness of mobile app–based stress-management program: a randomized controlled trial. Int J Environ Res Public Health.

[32] Birney AJ, Gunn R, Russell JK, Ary DV (2016). MoodHacker mobile web app with email for adults to self-manage mild-to-moderate depression: randomized controlled trial. JMIR Mhealth Uhealth.

[33] Mistretta EG, Davis MC, Temkit M, Lorenz C, Darby B, Stonnington CM (2018). Resilience training for work-related stress among health care workers: results of a randomized clinical trial comparing in-person and smartphone-delivered interventions. J Occup Environ Med.

[34] Smith EN, Santoro E, Moraveji N, Susi M, Crum AJ (2020). Integrating wearables in stress management interventions: promising evidence from a randomized trial. Int J Stress Manag.

[35] Hsieh HF, Huang IC, Liu Y, Chen WL, Lee YW, Hsu HT (2020). The effects of biofeedback training and smartphone-delivered biofeedback training on resilience, occupational stress, and depressive symptoms among abused psychiatric nurses. Int J Environ Res Public Health.

[36] Sasaki N, Imamura K, Tran TTT (2021). Effects of smartphone-based stress management on improving work engagement among nurses in Vietnam: secondary analysis of a three-arm randomized controlled trial. J Med Internet Res.

[37] Ly KH, Asplund K, Andersson G (2014). Stress management for middle managers via an acceptance and commitment-based smartphone application: a randomized controlled trial. Internet Interv.

[38] Deady M, Glozier N, Calvo R (2022). Preventing depression using a smartphone app: a randomized controlled trial. Psychol Med.

[39] Weber S, Lorenz C, Hemmings N (2019). Improving stress and positive mental health at work via an app-based intervention: a large-scale multi-center randomized control trial. Front Psychol.

[40] Taylor H, Cavanagh K, Field AP, Strauss C (2022). Health care workers’ need for Headspace: findings from a multisite definitive randomized controlled trial of an unguided digital mindfulness-based self-help app to reduce healthcare worker stress. JMIR Mhealth Uhealth.

[41] Taylor CB, Fitzsimmons-Craft EE, Graham AK (2020). Digital technology can revolutionize mental health services delivery: the COVID-19 crisis as a catalyst for change. Int J Eat Disord.

[42] Robbins R, Weaver MD, Quan SF (2022). Evaluating the impact of a sleep health education and a personalised smartphone application on sleep, productivity and healthcare utilisation among employees: results of a randomised clinical trial. BMJ Open.

[43] Gnanapragasam SN, Tinch-Taylor R, Scott HR (2023). Multicentre, England-wide randomised controlled trial of the “Foundations” smartphone application in improving mental health and well-being in a healthcare worker population. Br J Psychiatry.

[44] Pratt EH, Hall L, Jennings C (2023). Mobile mindfulness for psychological distress and burnout among frontline COVID-19 nurses: a pilot randomized trial. Ann Am Thorac Soc.

[45] Deady M, Collins DAJ, Lavender I (2023). Selective prevention of depression in workers using a smartphone app: randomized controlled trial. J Med Internet Res.

[46] Lee YJ (2023). Effects of a mobile health intervention on activities of stress self-management for workers. Work.

[47] Huberty JL, Espel-Huynh HM, Neher TL, Puzia ME (2022). Testing the pragmatic effectiveness of a consumer-based mindfulness mobile app in the workplace: randomized controlled trial. JMIR Mhealth Uhealth.

[48] Yoon SI, Lee SI, Suh HW, Chung SY, Kim JW (2022). Effects of mobile mindfulness training on mental health of employees: A CONSORT-compliant pilot randomized controlled trial. Medicine (Baltimore).

[49] Jukic T, Ihan A, Strojnik V, Stubljar D, Starc A (2020). The effect of active occupational stress management on psychosocial and physiological wellbeing: a pilot study. BMC Med Inform Decis Mak.

[50] Lappalainen P, Kaipainen K, Lappalainen R (2013). Feasibility of a personal health technology-based psychological intervention for men with stress and mood problems: randomized controlled pilot trial. JMIR Res Protoc.

[51] Collins DAJ, Harvey SB, Lavender I, Glozier N, Christensen H, Deady M (2020). A pilot evaluation of a smartphone application for workplace depression. Int J Environ Res Public Health.

[52] Hamamura T, Suganuma S, Ueda M, Mearns J, Shimoyama H (2018). Standalone effects of a cognitive behavioral intervention using a mobile phone app on psychological distress and alcohol consumption among Japanese workers: pilot nonrandomized controlled trial. JMIR Ment Health.

[53] Jesuthasan J, Low M, Ong T (2022). The impact of personalized human support on engagement with behavioral intervention technologies for employee mental health: an exploratory retrospective study. Front Digit Health.

[54] Counson I, Bartholomew A, Crawford J (2021). Development of the Shift smartphone app to support the emotional well-being of junior physicians: design of a prototype and results of usability and acceptability testing. JMIR Form Res.

[55] Golden EA, Zweig M, Danieletto M (2021). A resilience-building app to support the mental health of health care workers in the COVID-19 era: design process, distribution, and evaluation. JMIR Form Res.

[56] Gradito Dubord MA, Forest J, Balčiūnaitė LM, Rauen E, Jungert T (2022). The power of strength-oriented feedback enlightened by self-determination theory: a positive technology-based intervention. J Happiness Stud.

[57] Baek JH, Kim JH, Oh S, Kim JY, Baik S (2018). Smart stress care: usability, feasibility and preliminary efficacy of fully automated stress management application for employees. Psychiatry Investig.

[58] Crivelli D, Fronda G, Venturella I, Balconi M (2019). Stress and neurocognitive efficiency in managerial contexts: a study on technology-mediated mindfulness practice. Int J Workplace Health Manag.

[59] Deady M, Johnston D, Milne D (2018). Preliminary effectiveness of a smartphone app to reduce depressive symptoms in the workplace: feasibility and acceptability study. JMIR Mhealth Uhealth.

[60] Sanatkar S, Counson I, Mackinnon A, Bartholomew A, Glozier N, Harvey S (2022). Preliminary investigation of Shift, a novel smartphone app to support junior doctors’ mental health and well-being: examination of symptom progression, usability, and acceptability after 1 month of use. J Med Internet Res.

[61] Gbeddy GA (2021). Using mindfulness-based practice to reduce work-related stress and burnout among psychiatric nurses [Dissertation].

[62] Pace TWW, Zeiders KH, Cook SH (2022). Feasibility, acceptability, and preliminary efficacy of an app-based meditation intervention to decrease firefighter psychological distress and burnout: a one-group pilot study. JMIR Form Res.

[63] Ravalier JM (2022). Co-design, delivery, and evaluation of wellbeing initiatives for NHS staff: the HOW (healthier outcomes at work) NHS project. Int J Environ Res Public Health.

[64] Ravalier JM, Wainwright E, Smyth N, Clabburn O, Wegrzynek P, Loon M (2020). Co-creating and evaluating an app-based well-being intervention: the HOW (Healthier Outcomes at Work) Social Work Project. Int J Environ Res Public Health.

[65] Morris ME, Kathawala Q, Leen TK (2010). Mobile therapy: case study evaluations of a cell phone application for emotional self-awareness. J Med Internet Res.

[66] Muuraiskangas S, Harjumaa M, Kaipainen K, Ermes M (2016). Process and effects evaluation of a digital mental health intervention targeted at improving occupational well-being: lessons from an intervention study with failed adoption. JMIR Ment Health.

[67] Deady M, Glozier N, Collins D (2020). The utility of a mental health app in apprentice workers: a pilot study. Front Public Health.

[68] Xu HG, Tuckett A, Kynoch K, Eley R (2021). A mobile mindfulness intervention for emergency department staff to improve stress and wellbeing: a qualitative study. Int Emerg Nurs.

[69] de Korte EM, Wiezer N, Janssen JH, Vink P, Kraaij W (2018). Evaluating an mHealth app for health and well-being at work: mixed-method qualitative study. JMIR Mhealth Uhealth.

[70] Stoyanov SR, Hides L, Kavanagh DJ, Wilson H (2016). Development and validation of the user version of the Mobile Application Rating Scale (uMARS). JMIR Mhealth Uhealth.

[71] Grier RA, Bangor A, Kortum P, Peres SC (2013). The system usability scale: beyond standard usability testing. Proc Hum Factors Ergon Soc Annu Meet.

[72] Wilson CJ, Deane FP, Ciarrochi J, Rickwood D (2005). General Help Seeking Questionnaire (GHSQ). APA PsycTests.

[73] Kroenke K, Spitzer RL (2002). The PHQ-9: a new depression diagnostic and severity measure. Psychiatr Ann.

[74] Spitzer RL, Kroenke K, Williams JBW, Löwe B (2006). A brief measure for assessing generalized anxiety disorder: the GAD-7. Arch Intern Med.

[75] Cohen S, Kamarck T, Mermelstein R (1983). A global measure of perceived stress. J Health Soc Behav.

[76] Topp CW, Østergaard SD, Søndergaard S, Bech P (2015). The WHO-5 Well-Being Index: a systematic review of the literature. Psychother Psychosom.

[77] Tennant R, Hiller L, Fishwick R (2007). The Warwick-Edinburgh Mental Well-Being Scale (WEMWBS): development and UK validation. Health Qual Life Outcomes.

[78] Phillips EA, Gordeev VS, Schreyögg J (2019). Effectiveness of occupational e-mental health interventions: a systematic review and meta-analysis of randomized controlled trials. Scand J Work Environ Health.

[79] Cameron G, Mulvenna M, Ennis E (2025). Effectiveness of digital mental health interventions in the workplace: umbrella review of systematic reviews. JMIR Ment Health.

[80] Iribarren SJ, Akande TO, Kamp KJ, Barry D, Kader YG, Suelzer E (2021). Effectiveness of mobile apps to promote health and manage disease: systematic review and meta-analysis of randomized controlled trials. JMIR Mhealth Uhealth.

[81] Bernard RM, Toppo C, Raggi A (2022). Strategies for implementing occupational eMental health interventions: scoping review. J Med Internet Res.

[82] Agarwal S, LeFevre AE, Lee J (2016). Guidelines for reporting of health interventions using mobile phones: mobile health (mHealth) evidence reporting and assessment (mERA) checklist. BMJ.

[83] Yardley L, Spring BJ, Riper H (2016). Understanding and promoting effective engagement with digital behavior change interventions. Am J Prev Med.

[84] Torous J, Nicholas J, Larsen ME, Firth J, Christensen H (2018). Clinical review of user engagement with mental health smartphone apps: evidence, theory and improvements. Evid Based Ment Health.

[85] Zhang R, Nicholas J, Knapp AA (2019). Clinically meaningful use of mental health apps and its effects on depression: mixed methods study. J Med Internet Res.

[86] Tsantila F, Coppens E, De Witte H (2023). Developing a framework for evaluation: a theory of change for complex workplace mental health interventions. BMC Public Health.

[87] Damschroder LJ, Reardon CM, Opra Widerquist MA, Lowery J (2022). Conceptualizing outcomes for use with the consolidated framework for implementation research (CFIR): the CFIR outcomes addendum. Implement Sci.

[88] Greenhalgh T, Wherton J, Papoutsi C (2017). Beyond adoption: a new framework for theorizing and evaluating nonadoption, abandonment, and challenges to the scale-up, spread, and sustainability of health and care technologies. J Med Internet Res.

[89] Eysenbach G, CONSORT-EHEALTH Group (2011). CONSORT-EHEALTH: Improving and standardizing evaluation reports of web-based and mobile health interventions. J Med Internet Res.

