# Supplementary material for: Mental Health Apps Implemented in the Workplace: Scoping Review of Trends and Gaps in Evaluation Research
Source: JMIR Mhealth Uhealth. 2026 Mar 31;14:e57046. doi: 10.2196/57046 (PMC13038184; doi:10.2196/57046)
Supplement: Multimedia Appendix 1 [file mhealth-v14-e57046-s001.docx]

**Scoping Review Search Strategy**

We searched the following electronic databases: PsycINFO, Embase, Medline, Cochrane database, OVID Healthstar, and OVID Emcare for peer reviewed studies from January 2000 until August 2023. A comprehensive search strategy including search terms, subject headings and syntax requirements for the databases was adapted from a previous systematic review of digital mental health interventions for workplace and was modified in consultation with a health science librarian. The search was conducted from May to August 2023.

Key terms:

1. Employment/
2. employment.ti,ab.
3. (job or worker or workplace or employee* or occupation* or manager).ti,ab.
4. 1 or 2 or 3
5. mhealth.mp.
6. e-mental health.mp.
7. smartphone.ti,ab.
8. ((mobile or digital) adj2 (app or application)).ti,ab.
9. 5 or 6 or 7 or 8
10. Mental Disorders/
11. Mental Health/
12. ((mental* or mood or affect*) adj2 (disorder* or health or illness)).ti,ab.
13. anxiety.ti,ab.
14. wellbeing.mp.
15. depress*.ti,ab.
16. mindfullness.mp.
17. 10 or 11 or 12 or 13 or 14 or 15 or 16
18. 4 and 9 and 17
