# Supplementary material for: Mental Health Apps Implemented in the Workplace: Scoping Review of Trends and Gaps in Evaluation Research
Source: JMIR Mhealth Uhealth. 2026 Mar 31;14:e57046. doi: 10.2196/57046 (PMC13038184; doi:10.2196/57046)
Supplement: Multimedia Appendix 2 [file mhealth-v14-e57046-s002.docx]

Appendix 2. Study evaluation data extraction table

| **Intervention Name** | **Methodology (Research Design, Sample Size & Measurement Timepoints)** | **Key Variables Evaluated** | | |
| --- | --- | --- | --- | --- |
|  |  | **App Engagement** | **Impact Evaluation** | **Process of Implementation** |
| **Unstructured Self-Guided** | | | | |
| ***e-pD-Work intervention*** [16] | **Design:** RCT Protocol  **Estimated Sample size:** 720  **Measurement Timepoints:** Baseline, follow-up (3-months, 6-months, 9-months, 12-months) | Acceptability and satisfaction with the interventions (e-Health Impact Questionnaire) | **Mental distress:**  Composite International Diagnostic Interview (CIDI), Depressive symptoms (PHQ-9), Anxiety (GAD-7)  **Mental Health:**  Quality of life (SF-12)  **Work-related:**  Satisfaction with paid work (adapted Job Content Instrument) | Cost-effectiveness, Cost-utility (QALYs measured by EuroQol-5D) |
| ***The Healthy Minds Programs*** [28] | **Design:** RCT  **Sample Size:** 666  **Measurement Timepoints:** Baseline, 1-Week, 2-Week, 3-Week, 4-Week, and 3-month Follow-up |  | **Mental distress:**  Perceived Stress (PSS, Depression & Anxiety (Patient Reported Outcomes Measuring Scale)  **Mental health:**  Wellbeing (WHO-5), Awareness (Subscale of the Five Facet Mindfulness Questionnaire), Self-Compassion, Social Connection (NIH Toolbox Loneliness Questionnaire), Purpose (Meaning in Life Questionnaire)  **Other:**  Insight Skill (Drexel Defusion Scale), Perseverative Thinking |  |
| ***Stress Management app*** (No official name) [31] | **Design:** RCT  **Sample Siz**e: 56 nurses (Intervention=26; Control=30)  **Measurement Timepoints**: Pre and Post-test (4 weeks) | App Satisfaction (researcher developed questions assessing pros and cons) | **Mental distress:**  Stress (PSS), Depression (PHQ-9), Anxiety (GAD-7)  **Mental health:**  Wellbeing (WHO-5), Self-efficacy (self-report)  **Work-related:**  Job Stress (Korean Occupational Stress Scale), Perceived working conditions (Korean-Emotional Labor scale) |  |
| ***Smartphone-Delivered Biofeedback Training*** [35] | **Design:** RCT [3 groups:- Biofeedback Training (BT), Smartphone Delivered Biofeedback Training (SDBT), and Control]  **Sample Size:** 135  **Measurement Timepoints:** Baseline and Post-intervention (6-weeks) |  | **Mental distress:**  Depressive symptoms (Centre for Epidemiologic Studies Depression scale (CES-D)  **Mental health:**  Resilience Scale  **Work-related:**  Occupational Stress Indicator-2 (OSI-2)  **Other:**  Simplified health scale,  Physiological Measurements (electrocardiogram) |  |
| ***Lift Intervention*** [44] | **Design:** Pilot RCT  **Sample Size:** 102  **Measurement timepoints:** Baseline and post-intervention (4-weeks) | App use (Intervention completion, frequency of usage) | **Mental distress:**  Depression (PHQ-9), Anxiety (GAD-7), Burnout (MBI) |  |
| ***InMind*** [48] | **Design:** RCT  **Sample size:** 45  **Measurement Timepoints:** Baseline and Four-weeks (post-intervention, Eight Weeks (Follow-up) |  | **Mental distress:**  Perceived Stress (PSS)  **Mental Health:**  Wellbeing (Concise Measure of Subjective Wellbeing), Mibyeong Index) |  |
| ***24alife App*** [49] | **Design:** Pilot RCT  **Sample size:** 17  **Measurement Timepoints:** Baseline, 1-month, 2-months and 3-months | App usage | **Mental Distress:**  Anxiety (*State-Trait Anxiety Inventory*), Biological stress markers (Blood tests), Burnout (Maslow burnout inventory). Fatigue (*Modified Fatigue Impact Scale* (MFIS))  **Mental health:**  Life Satisfaction (*Satisfaction with Life Scale)*  **Other:**  Cardiovascular fitness levels (Rockport Fitness Walking Test), Muscle flexibility (standardized Sit and Reach Test), Muscle strength test (Sit up test), General Health (*General Health Questionnaire* (GHQ)) |  |
| ***A Self-Record App*** [52] | **Design:** Pilot Non-randomized Controlled Trial  **Sample size:** 1396  **Measurement Timepoints:** Baseline and 4-Weeks |  | **Mental distress:**  Mood (Japanese Negative Mood Regulation Scale), Distress (Japanese Version of Kessler Distress Scale K6), Anxiety (State Trait Anxiety Inventory), Alcohol use (The Daily Drinking Questionnaire)  **Mental health:**  Wellbeing (WHO-5-Japanese) |  |
| ***The Wellness Hub App*** [55] | **Design:** Pre/Post Non-randomized experimental design  **Sample Size:** 231  **Measurement Timepoints:** Continuous | App usage (App downloads, re-opens, survey completion) | **Mental distress:**  Ecological Mood Assessments, Depression (PHQ-8), Anxiety (GAD-2), Psychological Trauma (PCL-5), Alcohol Use (AUDIT C)  **Mental health:**  Resilience (CD RISC), Wellbeing (WHO-5), Spiritual Struggle |  |
| ***Listen Leon*** [56] | **Design:** Pre/Post Non-randomized Experimental design  **Sample Size:** 198  **Measurement Timepoints:** Baseline and 4-Weeks | Not reported | **Mental health:**  Psychological Wellbeing Scale,  Basic Psychological Needs Scale  **Work-Related:**  Multidimensional Work Motivation Scale  **Other:**  Strengths use and deficit correction scale |  |
| ***Stress Management App*** [57] | **Design:** Pre/Post Non-Experimental  **Sample size**: 68  **Measurement Timepoints:** Baseline, and 4 weeks | App Use (frequency, duration),  Satisfaction with usability (5-point rating scale) | **Mental distress:**  Perceived Stress (Brief encounter psychosocial instrument -Korean), Depressive Symptoms (Center for epidemiologic studies depression scale (CES-D)), Alcohol use (Korean version of the alcohol use disorders identification test (AUDIT))  **Mental health:**  Social Readjustment rating scale, Resilience (Korean resilience quotient)  **Work-related:**  Occupational stress scale (Korean)  **Other:**  Exercise and Diet patterns, Smoking, Type A personality (Framingham type A behavior pattern) |  |
| ***Healthier Outcomes at Work (HOW) app*** [63] | **Design:** Pre/Post Non-randomized study  **Sample Size:** 786 pre-survey; 129 post-survey  **Measurement Timepoints:** Baseline follow-up surveys (1-year from baseline) | Not reported | **Mental distress:**  Perceived Stress Scale (PSS)  **Mental health:**  Warwick-Edinburgh Mental Well-being scale  **Work-related:**  Utrecht Work Engagement Scale, Working conditions (Management Standards Indicator Tool, Presenteeism (1 item), Job Satisfaction (Self-report) |  |
| ***HOWApp*** [64] | **Design:** Mixed-method pre/post, non randomized  **Sample size:**  Interviews=19,  Focus Groups=8 (4 groups),  Pre survey=503,  Post survey=154  **Measurement Timepoints:**  Baseline & follow-up surveys, evaluation interviews (6-months implementation period; 1-year from baseline) | Number of downloads, Feedback from app users | **Mental health:**  General Health Questionnaire  **Work-related:**  Working conditions (Management Standards Indicator Tool (MSIT) |  |
| ***The Mood Map*** [65] | **Design:** Mixed-method design (Case Studies & Experience sampling data)  **Sample**: Individuals=5  **Measurement Timepoints:** 4 weekly interviews | Usage patterns (number of mood map entries) | **Mental distress:**  Single-dimension mood scales for anger, anxiety, happiness, and sadness built within the app. |  |
| ***Ovia*** [66] | **Design:** Mixed-method  **Sample size**  Surveys=43  Interviews=13  **Measurement Timepoints:** Baseline, 2 months, and 4 months | App usage patterns,  Motivation to use the app (14-item Self-Regulation Questionnaire) | **Mental Health:**  Wellbeing **(**84-item work-related  well-being questionnaire), Satisfaction with Life Scale, Mindfulness (14-item scale)  **Work-Related:**  Occupational health and well-being (1-item), Utrecht Work Engagement Scale,Work-Related Acceptance and Action Questionnaire (WAAQ) | Implementation process design based on Nielsen and Abildgaard’s framework (Initiation, Screening, Action Plan, Implementation and Evaluation of Effects) |
| ***Brightr App*** [73] | **Design: Qualitative**  **Sample size:**  User Interviews (n=22)  User focus groups (n=15)  Expert focus group (n=6)  **Measurement Timeline:** Baseline and post-intervention (3-months) | General impressions, Quality Assessment (Information, Service, Usefulness, Outcomes expectations) |  | Perspectives on drivers and barriers (Focus group & Interview with app users & experts) |
| **Structured Self-Guided** | | | | |
| ***Unmind App*** [18] | **Design:** RCT Protocol  **Estimated Sample size:** 300  **Measurement Timepoints:** Baseline, post-intervention (3-weeks), follow-up (7-weeks) | Duration of app use  Feedback (MARS) | **Mental distress:**  Mood (PHQ), Anxiety (GAD-7)  **Mental health**:  Unmind Index, Warwick-Edinburgh Mental Wellbeing Scale (WEMWBS) |  |
| ***Unmind App*** [41] | **Design:** Pilot RCT  **Sample Size:** 2003  **Measurement Timepoints:** Baseline, post-intervention (21-days) and follow-up (28 days) | Module completion rates, Feedback (MARS) | **Mental distress:**  Symptoms of Depression (PHQ-8), Anxiety (GAD-7)  **Mental health:**  Unmind Index, Wellbeing (WEMWBS)  **Work-related:**  Work productivity and Impairment Scale (WPAI) |  |
| ***The Skills For Life Adjustment and Resilience (SOLAR-m)*** [20] | **Design:** RCT Protocol  **Estimated Sample size:** 240  **Measurement Timepoints:** Baseline, post-intervention (7-Weeks), and follow-up (3-month) |  | **Mental distress:**  Anxiety (subscore of the HADS), Depression (sub score of the HADS), Trauma (PCL-5),  Work Family Conflict scale  **Other:**  Treatment Expectations (Credibility Expectancy Questionnaire (CEQ-II) |  |
| ***WorkingWell*** [22] | **Design:** Mixed-method Protocol  **Estimated Sample size:** 40  **Measurement Timepoints:** Baseline, post-intervention (8-weeks) | **App usage** (daily navigation within the app features), **Perceived usability** (Systems Usability Scale) | **Work-related:**  Motivation (Job Match Survey),  Work-related self-efficacy  **Other:**  Social Support (18-item Medical Outcomes Study Social Support Survey) |  |
| ***EMPOWER*** [23] | **Design:** RCT Protocol – Stepped wedge cluster RCT  **Estimated Sample size:** 874  **Measurement Timepoints:** Baseline, pre & post intervention (at 7 weeks) & 3-month follow-up | **App usage** (# logins, frequency & duration of use) | **Mental distress:**  Depression (PHQ-9), anxiety (GAD-7),  Perceived stress (PSS-4), patient health questionnaire (PHQ-15), severity of insomnia (1 item)  **Mental health:**  Well-being (WHO-5),Mental health quality of life (MHQoL),EuroQol questionnaire (EQ-5D-5L)  **Work-related:**  Presenteeism, absenteeism (from iPCQ productivity cost questionnaire)  **Other:**  Physical activity level (IPAQ),  Physical symptoms (PHQ-15 somatic scale), Healthcare utilization (iMCQ) | Cost-effectiveness & cost utility analysis |
| ***WEDiary***  [24] | **Design:** RCT  **Sample size:** 600  **Measurement Timepoints:** Baseline, post (2 weeks), 3-week follow-up | **App usage** (Downloads, task completion, usage over time) | **Work-related:**  Work Utrecht Work Engagement scale |  |
| ***Holidaily*** [25] | **Design:** RCT Protocol  **Estimated Sample size:** 77  **Measurement Timepoints:** Six timepoints -Two weeks pre-vacation, last working day, mid vacation, first working day, two weeks post-vacation, four weeks post-vacation | User experience (Mobile App Rating Scale) | **Mental distress:**  Depression (PHQ-8), Insomnia (Insomnia Severity Index), Emotional exhaustion (Maslach Burnout Inventory)  **Mental health:**  Satisfaction of psychological needs (DRAMMA questionnaire)  **Work-related:**  Work-related rumination questionnaire |  |
| ***Headspace*** [26] | **Design:** RCT Protocol  **Estimated Sample size:** 2000  **Measurement Timepoints:** Baseline, Midpoint (4-weeks), post-intervention (8-weeks), 4 months follow-up |  | **Mental distress:**  Perceived stress scale (PSS),  Bergen Burnout Inventory  **Mental health:**  Mindful Attention Awareness Scale  **Work-related:**  Utrecht Work Engagement Scale, Job Strain (Siegrist Job Strain Scale) |  |
| ***Headspace*** [27] | **Design:** RCT  **Sample Size:** 148  **Measurement Timepoints:** Pre, post (4 weeks) & 3-month follow-up | Self-report of app use | **Mental distress:**  Perceived Stress scale, Maslach Burnout Inventory  **Mental health:**  Warwick-Edinburgh Mental Well-being Scale, Mindfulness Attention Awareness Scale |  |
| ***Headspace*** [40] | **Design:** RCT  **Sample Size:** 2182  **Measurement Timeline:** Baseline, 1.5 months, 4.5 months | Self-reported app use, user feedback | **Mental distress:**  Depression (DASS-21), Burnout (Maslach Burnout Inventory), Worry (Penn State Worry Questionnaire), Rumination (Brooding subscale of the Ruminative Response scale)  **Mental Health:**  Wellbeing (Warwick Edinburgh Mental Wellbeing Scale), Mindfulness (Five facets of Mindfulness Questionnaire), Self-Compassion (Self-Compassion Short Form)  **Work-related:**  Sickness Absence |  |
| ***Headspace*** [68] | **Design:** Descriptive Qualitative Study  **Sample Size:** 24  **Measurement Timepoints:** Interviews at post-intervention (4-weeks) | Self-reported app use, user feedback | Perceived impact | Perceived barriers and facilitators to app use |
| ***BetterLife*** [29] | **Design:** RCT  **Sample size:** 126  **Measurement Timepoints:** Baseline and 10-Weeks |  | **Mental distress:**  Depression (BDI-II), Anxiety (BAI), Perceived stress (PSS)  **Mental health:**  Quality of Life (WHOQOL)  **Work-related:**  Work Engagement (Utrecht Work Engagement Scale) |  |
| ***Well-Being Mobile App*** [30] | **Design**: RCT  **Sample size:** 490  **Measurement Timepoints:** Baseline, 1-month midpoint, post-intervention (2-months) | Adherence rates (completion of prescribed app use) | **Mental distress:**  Perceived Stress Scale (PSS)  **Mental health:**  Well-being (WHO Well-Being Index)  Subjective ratings of well-being |  |
| ***The MoodHacker*** [32] | **Design:** RCT  **Sample size:** 300 **Measurement Timepoints:** Baseline, follow-up at 6 weeks, and 10 weeks | Usage Patterns (Number of logins, duration of app use), User Satisfaction (System Usability Scale) | **Mental distress:** Depression symptoms (PHQ-9), Negative Cognitions (Automatic Thoughts Questionnaire)  **Work-related:**  Performance (Work Limitations Questionnaire),  Absenteeism (WLQ Work Absence), Workplace Outcome Suite (WOS)  **Other:**  Mental health knowledge (14 multiple-choice items developed by authors),  Behavioral activation scale |  |
| ***Smartphone Resiliency Training*** [33] | **Design: RCT**  (3 groups: Control, In-person Training, and Smartphone)  **Sample size**: 60  **Measurement Timepoints:**  Baseline, Follow up at 6-weeks and 3-months |  | **Mental distress:**  Depression, Anxiety, and Stress Scale (DASS-21)  **Mental health:**  Wellbeing (WHO-5), Self-compassion Scale, Compassion for others scale, Daily Affect, Relationship Quality  **Work-related:**  Work related burnout (MBI-Human Services Survey)  **Other:**  Sleep monitoring |  |
| ***ABC Stress Management*** [36] | **Design:** RCT (Control + 2 Intervention groups)  **Sample size**: 951  **Measurement Timepoints:** Baseline, post-intervention (3-months) and follow-up (7-months) |  | **Work-related:**  Utrecht Work Engagement Scale |  |
| ***Acceptance and Commitment Therapy (ACT) based App*** [37] | **Design:** RCT  **Sample size**: 73 **Measurement Timeline:** Baseline and post-intervention (6-weeks) | Usage (self-report), Adherence (minimum of two modules completed), attrition rates | **Mental distress:**  Perceived Stress (PSS-14, PSS-10)  **Mental health:**  General Health (GHQ-12)  **Work-related:**  Leadership effectiveness (Multifactor Leadership Questionnaire) |  |
| ***HeadGear*** [38] | **Design:** RCT (Intervention and Attention Control App)  **Sample size**: 2271 **Measurement Timeline**: baseline, post-intervention (5 weeks after baseline), 3- and 12-month follow-up. | Usage based on number of challenges completed | **Mental distress:**  Depression symptoms (Patient Health Questionnaire-9 (PHQ-9)), Anxiety (Generalized Anxiety Disorder scale (GAD-2))  **Mental health:**  Resilience Connor-Davidson Resilience Scale (CD-RISC10), Wellbeing (WHO Wellbeing Index (WHO-5))  **Work-related:**  Work performance (Health and Work Performance Questionnaire) |  |
| ***HeadGear*** [17] | **Design:** RCT  **Sample size:**  Stage 2=84  **Measurement Timepoints:** Baseline, post-intervention (5-weeks), follow-up (3-months) | Usage data: number of logins, time spent in the app, responses to exercises | **Mental distress:**  Depression Symptoms (PHQ-9), Anxiety (GAD-2)  **Mental health:**  WHO Well-being index,  Resilience (Connor-Davidson Resilience scale)  **Work-related:**  WHO Health and Work Performance Questionnaire), Absenteeism (2 questions) |  |
| ***Anchored App*** (Adapted from HeadGear App) [45] | **Design:** RCT  **Sample Size:** 2112  **Measurement Timepoints:** Baseline, One month follow-up, Three months follow-up and 6-month follow-up | App Use (Number of challenges/ modules completed) | **Mental distress:**  Symptoms of Depression (PHQ-9), Anxiety (GAD-7), Perceived Stress (PSS)  **Mental health:**  Wellbeing (WHO-5), Resilience (BRS)  **Work-related:**  Work-related Burnout (Copenhagen Burnout Inventory), Work Performance (Health and Work Performance), Absenteeism (Number of sickness absences over last 28 days) |  |
| ***Anchored App*** (Adapted from HeadGear App) [51] | **Design**: Pilot RCT  **Sample size:** 81  **Measurement Timepoints:** Baseline, post-intervention (5 weeks) | App usage (number of challenges completed),  App feedback (Mobile Application Rating Scale (MARS)) | **Mental distress:**  Depressive symptoms (PHQ-9), Anxiety symptoms (GAD-7), Alcohol use (self-report)  **Mental health:**  Resilience (Brief Resilience Scale (BRS)), Wellbeing (WHO-5)  **Work-related:**  Work-related Stress (Single-item Stress Question (SISQ), Work performance and absenteeism (items from the Health and Work Performance Questionnaire (HPQ)) |  |
| ***HeadGear Apprentice App*** [67] | **Design:** Mixed-Method  **Sample size:** 26 participants across four focus groups  **Measurement Timeline:** Baseline, post-intervention (5-weeks), and follow-up (3-months) | App usage data,  Adapted MARS Scale, Adapted version of the Usefulness, Satisfaction, and Ease questionnaire | **Mental distress:**  Depressive symptoms (PHQ-9), Anxiety (GAD-7)  **Mental health:**  Wellbeing (WHO-5)  **Work-related:**  Work performance (HPQ), Presenteeism (days present at work x absolute work productivity score) |  |
| ***Kelaa Mental Resilience App*** [39] | **Design:** RCT  **Sample size:** 532  (Intervention=210, Control= 322)  **Measurement Timeline:** Baseline, mid-intervention (2 weeks), post-intervention (4-weeks), follow-up (6-weeks) | App usage patterns (Number of times activity is tracked) | **Mental distress:**  Stress (two subscales General Stress), Sleep (Sleeping Troubles from the COPSOQ II)  **Mental health:**  Wellbeing (Warwick-Edinburgh Mental Wellbeing Scale), Resilience (13-item Resilience Scale)  **Work-related:**  Social Community at Work (subscale from the COPSOQ II), Work Productivity and Activity Impairment Questionnaire  **Other:**  Physical Health (SF-36 Version 2) |  |
| ***Foundations App*** [43] | **Design:** RCT  **Sample Size:** 1002  **Measurement Timepoints:** Baseline, 4-week and 8-week |  | **Mental distress:**  Anxiety (GAD-7)  **Mental health:**  General Health (GHQ-12), Resilience (Brief Resilience Scale), Wellbeing (Warwick Edinburgh Mental Wellbeing Scale)  **Work-related:**  Stanford Presenteeism Scale, Work and Social Adjustment Scale  **Other:**  Supports accessed (Pharmacological, psychological, other apps) |  |
| ***Mobile based Stress Management Intervention (mSMI)*** [46] | **Design:** RCT  **Sample Size:** 82  **Measurement Timepoints:** Baseline and Six-Weeks | Duration of use,  Feedback from app users | **Mental distress:**  Perceived Stress (PSS-10), Hospital Anxiety & Depression scale  **Work-related:**  Utrecht Work Engagement scale |  |
| ***Calm App*** [47] | **Design:** RCT  **Sample Size:** 1029  **Measurement Timepoints:** Baseline, every 2-weeks, and 8-weeks (post intervention) | Use over time (Average # of sessions and minutes of use per week) | **Mental distress:**  Mental Health (DASS-21)), Sleep (Insomnia Severity Index)  **Mental health:**  Resilience (BRS)  **Work-related:**  Work Productivity and Impairment Questionnaire, Productivity Cost (Computed with Human Capital Approach)  **Other:**  Medical Care Visits |  |
| ***P4Well*** [50] | **Design:** Pilot RCT; Intervention, waitlist control  **Sample size:** 24 **Measurement Timeline:** Pre and Post intervention (6-Months) | Perceived utility, acceptance and usefulness of different components (Questionnaire developed by researchers) | **Mental distress:**  Psychological symptoms (general  symptom index (GSI)), Depression (BDI)  **Mental health:**  Quality of Life (Visual Analogue Scale for mood, self-rated health, life satisfaction, self-confidence, and working ability), Psychological Flexibility and Experimental Avoidance (Acceptance and Action Questionnaire-2 (AAQ-2))  **Work-related:**  Burnout (Bergen Burnout Indicator) |  |
| ***Naluri App*** [53] | **Design:** Retrospective Study  **Sample Size:** 88  **Measurement Timepoints:** Baseline, 3-weeks | App usage (Number of features explored, Number of messages sent to coaches, Number of modules completed) | **Mental distress:**  Depression (DASS-21) |  |
| ***Shift App*** [60] | **Design:** Pre/Post Non-randomized Experimental design  **Sample Size:** 222  **Measurement Timepoints:** Baseline and Post-intervention (4-weeks) | Usage (Number of logins, activities, and activity time),  Acceptability (5-point Likert Scale) | **Mental distress:**  Depression Symptoms (PHQ-9), Anxiety (GAD-7)  **Work-related:**  Work and Social Functioning (5-item Work and Social Adjustment Scale), COVID-19 Safety Concerns (2-items)  **Other:**  General Help Seeking Questionnaire |  |
| ***Shift App*** [54] | **Design**: Pre/Post Non-randomized Pilot study  **Sample Size:** 22  **Measurement Timepoints:** Baseline and post-intervention (4-weeks) |  | **Mental distress:**  Depression (PHQ-9),  Anxiety (GAD-7)  **Other:**  Intentions of Help-Seeking |  |
| ***VA Mindfulness Coach App*** [61] | **Design:** Pre/Post Non-randomized design  **Sample Size:** 14  **Measurement Timepoints:** Baseline and Four-weeks | Feedback from app users | **Mental distress:**  Maslach Burnout Inventory |  |
| ***Smartphone-based Mediation App*** [62] | **Design:** Pre/Post Non-randomized Experimental design  **Sample Size:** 35  **Measurement Timepoints:** Baseline and post-intervention (16-days) | App Use | **Mental distress:**  Symptoms of Anxiety and Depression (Patient reported Outcomes Measurement System), Burnout (Burnout measure short version), Negative Affect (The Positive and Negative Affect Schedule)  **Other:**  Salivary Cortisol |  |
| **Adjunct to other interventions** | | | | |
| ***MATESmobile program*** [21] | **Design:** RCT Protocol  **Estimated Sample size:** 844  **Measurement Timepoints:** Baseline, post-intervention (8-weeks), follow-up (3,6 and 12-months) | Usage patterns (Number of times app opened, time spent in the app), App feedback (study author developed questions for feedback) | **Mental distress:**  Psychological distress (Kessler-6 instrument), Suicidal ideation and behaviours (Suicidal Behavior Questionnaire-Revised)  **Work-related:**  Psychosocial Safety Climate (PSC-12)  **Other:**  Suicide prevention literacy,  Help-seeking and help-offering (General Help-Seeking Questionnaire) |  |
| ***Spire Stone wearable device and mobile App*** [34] | **Design:** RCT  **Sample size:** 169 **Measurement Timepoints:** Baseline and post-intervention (4 weeks) | Real time respiratory data from Spire Stone device to measure physiological stress | **Mental distress:**  Stress (PSS), Mood and Anxiety symptoms (MASQ), Healthy Days-Anxious Days (Center for Disease  Control’s Healthy Days Core and Symptoms Modules), Positive and Negative Affect scale  **Mental health:**  CDC health-related quality of life |  |
| ***Dayzz*** [42] | **Design:** RCT  **Sample Size:** 1355  **Measurement Timeline:** Baseline, Monthly and Follow-up (10-Months) | Perceived benefit and feedback (Self-report on 5–point Likert Scale) | **Mental health:**  Sleep Diary (Self-report of Sleep), Sleep Quality (Pittsburgh Sleep Quality Index), Mood, Alertness and Energy (Visual Analogue Scale)  **Work-related:**  Absenteeism Performance and Productivity (WHO Health and Work Performance Questionnaire)  **Other:**  Motor vehicle crashes and near crashes (monthly self-report), Healthcare Utilization |  |
| ***Brain-sensing eyeglasses and a corresponding mobile App*** [58] | **Design:** Pre/Post nonrandomized  **Sample size:** 16  **Measurement Timepoints:** Pre and Post intervention (2-weeks) |  | **Mental distress:**  Perceived Stress scale, Mood (Profile of Mood States inventory) State-Trait Anxiety Inventory,  Physiological markers of stress  **Other:**  Cognitive abilities (MIDA battery), Neurocognitive efficiency (software Stroop-like task) |  |

^a^Classification of the mental health apps employed in each study was done by review authors based on the intervention description provided in the respective studies.

^b^Outcomes are organized by the three categories of evaluation approaches discussed in the review.
